# Neutrophil Immunomodulatory Activity of Natural Organosulfur Compounds

**DOI:** 10.3390/molecules24091809

**Published:** 2019-05-10

**Authors:** Igor A. Schepetkin, Liliya N. Kirpotina, Andrei I. Khlebnikov, Narayanaganesh Balasubramanian, Mark T. Quinn

**Affiliations:** 1 Department of Microbiology and Immunology, Montana State University, Bozeman, MT 59717, USA; schepetkin@yahoo.com (I.A.S.); Liliya.kirpotina@montana.edu (L.N.K.); 2 Kizhner Research Center, Tomsk Polytechnic University, Tomsk 634050, Russia; aikhl@chem.org.ru; 3 Faculty of Chemistry, National Research Tomsk State University, Tomsk 634050, Russia; 4 Department of Chemistry and Biochemistry, Montana State University, Bozeman, MT 59717, USA; ganesh@montana.edu

**Keywords:** organosulfur compounds, phosphatidylinositol-3 kinase, reactive oxygen species, 1,3-dithiane, neutrophil, immunomodulation

## Abstract

Organosulfur compounds are bioactive components of garlic essential oil (EO), mustard oil, *Ferula* EOs, asafoetida, and other plant and food extracts. Traditionally, garlic (*Allium sativum*) is used to boost the immune system; however, the mechanisms involved in the putative immunomodulatory effects of garlic are unknown. We investigated the effects of garlic EO and 22 organosulfur compounds on human neutrophil responses. Garlic EO, allyl propyl disulfide, dipropyl disulfide, diallyl disulfide, and allyl isothiocyanate (AITC) directly activated Ca^2+^ flux in neutrophils, with the most potent being AITC. Although 1,3-dithiane did not activate neutrophil Ca^2+^ flux, this minor constituent of garlic EO stimulated neutrophil reactive oxygen species (ROS) production. In contrast, a close analog (1,4-dithiane) was unable to activate neutrophil ROS production. Although 1,3-dithiane-1-oxide also stimulated neutrophil ROS production, only traces of this oxidation product were generated after a 5 h treatment of HL60 cells with 1,3-dithiane. Evaluation of several phosphatidylinositol-3 kinase (PI3K) inhibitors with different subtype specificities (A-66, TGX 221, AS605240, and PI 3065) showed that the PI3K p110δ inhibitor PI 3065 was the most potent inhibitor of 1,3-dithiane-induced neutrophil ROS production. Furthermore, 1,3-dithiane enhanced the phosphorylation of extracellular signal-regulated kinase 1/2 (ERK1/2), glycogen synthase kinase 3 α/β (GSK-3α/β), and cAMP response element binding (CREB) protein in differentiated neutrophil-like HL60 cells. Density functional theory (DFT) calculations confirmed the reactivity of 1,3-dithiane vs. 1,4-dithiane, based on the frontier molecular orbital analysis. Our results demonstrate that certain organosulfur compounds can activate neutrophil functional activity and may serve as biological response modifiers by augmenting phagocyte functions.

## 1. Introduction

Garlic and onion are important in the diet and have been reported to benefit human health [1]. For example, compounds in garlic and onions have been reported to exhibit antimicrobial, anticancer, anti-inflammatory, anti-asthmatic, antidiabetes, antithrombotic, and antiplatelet activities [2,3,4,5,6,7,8]. Organosulfur compounds are present in many plants, and their bioactive properties have benefitted folk and traditional medicine throughout the centuries [1,9]. *Allium* and *Ferula* species, and essential oils (EOs) isolated from these spp., are rich in organosulfur compounds [10,11,12]. Some sulfide derivatives in garlic oil are formed as transformation products of allicin during distillation [13]. However, the immunomodulatory effects of garlic extract, garlic EOs, and pure organosulfur compounds have not been extensively evaluated [14,15,16,17,18]. For example, garlic extract was found to be an inhibitor of leukocyte migration through endothelial cell monolayers [19]. The anti-inflammatory effect of garlic EO was associated with suppressed neutrophil infiltration into tissue and with lowered levels of certain soluble and cellular adhesion molecules generated under inflammatory conditions [17]. Approximately 100 organosulfur compounds have been identified in garlic EO from *Allium sativum*, with diallyl sulfide, diallyl disulfide, allyl methyl sulfide, and diallyl trisulfide as the principal compounds [20]. Moreover, novel sulfides called garlicnins have been isolated from *Allium sativum* L. and were shown to modulate macrophage activity [21,22,23]. However, the effects of volatile organosulfur compounds from garlic EO on neutrophil functions have not been thoroughly examined. 

Neutrophils are key components of the innate immune system and play an integral role in normal tissue homeostasis, although their dysregulation is thought to contribute to the pathogenesis of numerous chronic inflammatory diseases, infectious disorders, and certain autoimmune diseases [24,25]. Neutrophils are professional phagocytes and the final effector cells of innate immunity, with a primary role in the clearance of extracellular pathogens. They can directly interact with macrophages, dendritic cells, natural killer cells, T cells, and B cells in order to either potentiate or resolve both innate and adaptive immune responses [26]. Consequently, the identification of substances that can modulate neutrophils is of great interest, and it is well established that a wide range of plant-derived compounds exhibit beneficial pharmacological effects via their ability to modulate phagocyte functions [27,28]. Indeed, several plant-derived small molecules have been shown to exhibit immunomodulatory activity via the regulation of neutrophil function [11,29,30,31]. Recently, we found that *Ferula*-derived EOs containing high amounts of propenyl *sec*-butyl disulfides stimulated Ca^2+^ mobilization and activated reactive oxygen species (ROS) production in human neutrophils [11]. Onion extracts and garlic EO contain many organosulfur compounds, but only a few of them have been evaluated with phagocytic cells. For example, diallyl sulfide, diallyl disulfide, and diallyl trisulfide suppressed endotoxin-induced neutrophil infiltration and damage in rat intestines [32]. Moreover, diallyl disulfide decreased nitric oxide (NO) production, with a reduction in the levels of interleukins (IL)-6 and IL-1β produced by macrophages stimulated with lipopolysaccharide [33]. The differential efficacy of these major organosulfur compounds on the suppression of inducible NO-synthase (iNOS) expression and NO production by macrophages was related to the number of sulfur atoms [34,35,36]. 

In the present study, we evaluated the effect of garlic EO and pure organosulfur compounds on neutrophil functional activity. All of these compounds were previously reported as components of garlic EO or mustard, garlic and onion extracts. We showed that allyl-containing organosulfur compounds and 1,3-dithiane activated human neutrophils, resulting in increased [Ca^2+^]_i_ and/or the production of ROS. Given the critical role played by phagocytes in innate immunity against pathogens, our data support the possibility that allyl-containing organosulfur compounds and 1,3-dithiane could be effective therapeutic modulators of innate immune responses. 

## 2. Results and Discussion

### 2.1. Effect of Garlic EO and Organosulfur Compounds on Neutrophil Ca^2+^ Flux

Garlic EO and twenty-two commercially available organosulfur compounds were screened for immunomodulatory activity in human neutrophils. Some of the test compounds are found in both garlic extracts and EOs, whereas other compounds, such as allicin and ajoene, are present only in garlic extracts. The plant sources and chemical structures of the selected organosulfur compounds are shown in Table 1. 

**Figure 1:**
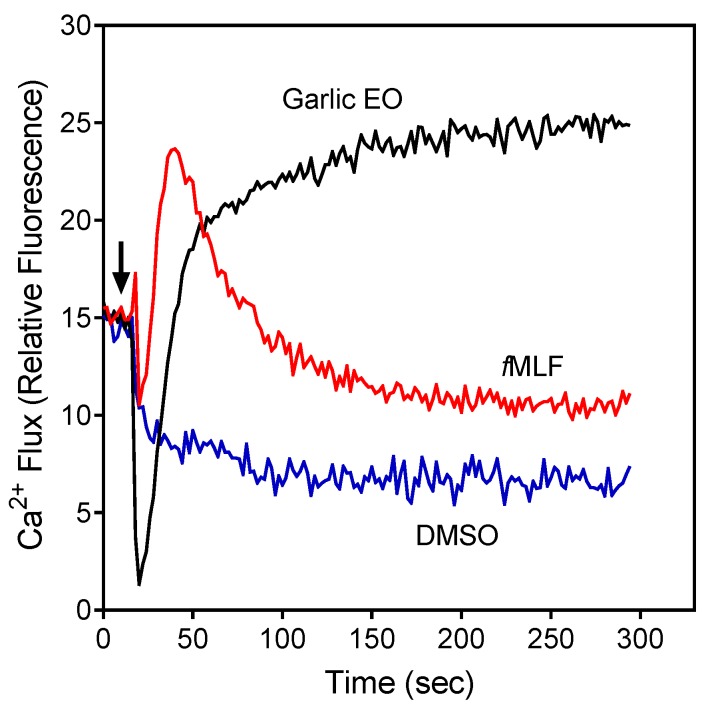
Effect of garlic EO on neutrophil Ca^2+^ mobilization in neutrophils. Human neutrophils were treated with 100 µg/mL garlic EO, 5 nM *f*MLF (positive control), or 1% dimethyl sulfoxide (DMSO; negative control), and [Ca^2+^]_i_ flux was monitored for the indicated times (the arrow indicates when the treatments were added). Data are from one experiment that is representative of three independent experiments.

The compounds were first evaluated for their effects on Ca^2+^ flux in human neutrophils, and we found that garlic EO activated neutrophil Ca^2+^ flux with an EC_50_ of 34.9 µg/mL. A representative kinetic curve is shown in Figure 1. We found that four of the 22 tested compounds (allyl propyl disulfide, dipropyl disulfide, diallyl disulfide, and allyl isothiocyanate (AITC)) directly activated neutrophil Ca^2+^ flux, with the most potent being AITC, a major component of mustard oil (Table 2). Thus, diallyl disulfide, a major component of garlic EO, may be a principal molecule responsible for activating neutrophil Ca^2+^ mobilization.

The compounds were also evaluated for their ability to inhibit *f*MLF-induced Ca^2+^ flux in neutrophils. The cells were pretreated with the organosulfur compounds for 30 min and then treated with 5 nM *f*MLF. Six compounds, including the four compounds shown above to have agonist activity (i.e., allyl propyl disulfide, dipropyl disulfide, diallyl disulfide, and AITC), as well as methyl propyl disulfide and allicin were found to be modest inhibitors of *f*MLF-induced Ca^2+^ mobilization, suggesting that pre-exposure of neutrophils to these compounds may desensitize them to activation by other agonists (Table 2). For example, garlic EO inhibited *f*MLF-induced Ca^2+^ flux in neutrophils with an IC_50_ of 14.7 µg/mL.

### 2.2. Effect of Garlic EO and Organosulfur Compounds on Neutrophil ROS Production

The organosulfur compounds and garlic EO were evaluated for their ability to modulate neutrophil ROS production. We found that garlic EO and three of the individual compounds (diallyl trisulfide, ajoene, and allicin) inhibited spontaneous ROS production by neutrophils. These compounds, as well as alliin, *N*-acetyl-*S*-allyl-L-cysteine, and *S*-allyl-L-cysteine, also inhibited phorbol-12-myristate-13-acetate (PMA)-stimulated ROS production by these cells (Table 2). Ajoene and allicin were the most potent inhibitors of neutrophil ROS production. It should be noted that during the process of steam distillation, allicin, which is a precursor of ajoene, is completely eliminated from garlic EO [44]. Thus, diallyl trisulfide, a major component compound of garlic EO, could be one of the main components responsible for ROS inhibition by garlic EO. 

Reactive oxygen species (ROS) production was monitored as L-012-dependent chemiluminescence (CL). EC_50_ and IC_50_ values were determined by nonlinear regression analysis of the dose-response curves, as described under Section 3.5, and are presented as the mean ±S.D. of three independent experiments. For the stimulation of ROS production, neutrophils were pretreated with phorbol-12-myristate-13-acetate (PMA) (100 nM). N.A., no inhibitory activity was found at concentrations up to 50 µM. ^*^Instead of inhibition, 1,3-dithiane directly activated neutrophil ROS production (see below).

**Figure 2:**
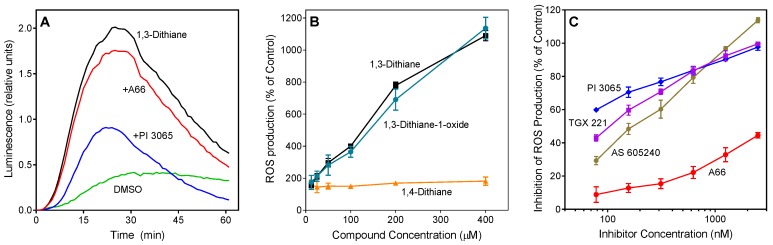
Effect of 1,3-dithiane, 1,4-dithiane, and 1,3-dithiane-1-oxide on human neutrophil ROS production. (**A**). Effect of phosphatidylinositol-3 kinase (PI3K) inhibitors on 1,3-dithiane-induced ROS production. Neutrophils were treated with 1,3-dithiane (200 μM), 1,3-dithiane (200 μM) in the presence of the indicated PI3K inhibitors A66 or PI 3065 (150 nM each), or DMSO (control), and L-012-dependent CL was monitored for 60 min. Representative of 3 independent experiments. (**B**). Concentration-dependent ROS production induced by 1,3-dithiane and 1,3-dithiane-1-oxide. Neutrophils were treated with the indicated concentrations of 1,3-dithiane, 1,4-dithiane, or 1,3-dithiane-1-oxide, and L-012-dependent CL was monitored for 60 min. ROS production monitored for 60 min is shown (% of control). (**C**). Concentration-dependent inhibition of 1,3-dithiane-induced ROS production by selected PI3K inhibitors. Neutrophils were treated with 1,3-dithiane (200 μM) or 1,3-dithiane (200 μM) in the presence of varying concentrations of the indicated PI3K inhibitors, and L-012-dependent CL was monitored for 60 min. Inhibition of ROS production monitored for 60 min is shown (% of control). The data in Panels B and C are presented as mean ±S.D. of triplicate samples from one experiment that is representative of three independent experiments.

In contrast to the ROS inhibition exhibited by several compounds (Table 2), 1,3-dithiane actually activated neutrophil ROS production. This compound stimulated ROS production with unimodal kinetics, which is similar to that observed for other phagocyte activating agents [45] (Figure 2A). Note that 1,3-dithiane-stimulated ROS production was completely inhibited by superoxide dismutase (SOD, 100 U/mL), and 1,3-dithiane had no effect on the ROS assay system in the absence of cells, indicating neutrophil NADPH oxidase activation rather than an artifact (data not shown). Although 1,4-dithiane (an analog of 1,3-dithiane) has not been reported to be present in plant extracts/EOs, it has been reported to be present in products of bacterial fermentation and boiled beef extract [46,47]. Thus, we also tested 1,4-dithiane under the same conditions and found that it had no effect on ROS production, demonstrating that the effects of 1,3-dithiane were specific for this compound (Figure 2B). Since 1,3-dithiane is efficiently oxidized by flavin monooxygenases into the corresponding sulfoxide enantiomer (*R*)-1,3-dithiane-1-oxide [48], we could not exclude that the biotransformation products of 1,3-dithiane might also activate neutrophils. Thus, we tested 1,3-dithiane-1-oxide and found that its activity was similar to 1,3-dithiane itself (Figure 2B), indicating that either 1,3-dithiane or its oxide could be responsible for the observed activation of neutrophil ROS production. To further investigate this issue, we incubated HL60 cells with 1,3-dithiane for 0, 1, and 5 h and evaluated the culture medium for oxidation products. Analysis of the cell lysates by gas chromatography–mass spectrometry (GC-MS) showed the *m*/*z* of the 1,3-dithiane-1-oxide (M^+^) ion to be 136.00. The electron impact (EI) mass spectrum also indicated the presence of trace amounts of 1,3-dithiane-1-oxide, but only after the 5 h incubation, and the identity of this compound was confirmed using a reference compound and the NIST 14 MS library embedded in the Agilent data analysis software (data not shown). Thus, neutrophil activation is primarily due to 1,3-dithiane, especially during the earlier treatment times evaluated in this study (0–60 min), whereas trace amounts of the oxidation product 1,3-dithiane-1-oxide could contribute to cell activation at much later times.

### 2.3. Effect of Phosphatidylinositol-3 Kinase (PI3K) Inhibitors

Because PI3K plays an important role in the regulation of ROS production by human neutrophils [49,50], we evaluated the effect of specific inhibitors of various PI3K isoforms on 1,3-dithiane-stimulated ROS production in neutrophils. Four PI3K inhibitors with different subtype specificities, including A-66, TGX 221, AS605240, and PI-3065 [51,52,53], were tested. PI-3065, a PI3K p110δ inhibitor, demonstrated the most potent inhibitory effect (IC_50_ = 0.03 ± 0.01 µM). The other inhibitors had lower activity, as follows: TGX 221 (PI3K-β inhibitor, IC_50_ = 0.10 ± 0.03 µM) > AS 605240 (PI3Kγ inhibitor, IC_50_ = 0.18 ± 0.04 µM) >> A66 (PI3K p110α inhibitor, IC_50_ = 3.9 ± 1.2 µM) (Figure 2A,C). 

### 2.4. Effect of 1,3-Dithiane on Protein Kinase Phosphorylation

**Figure 3:**
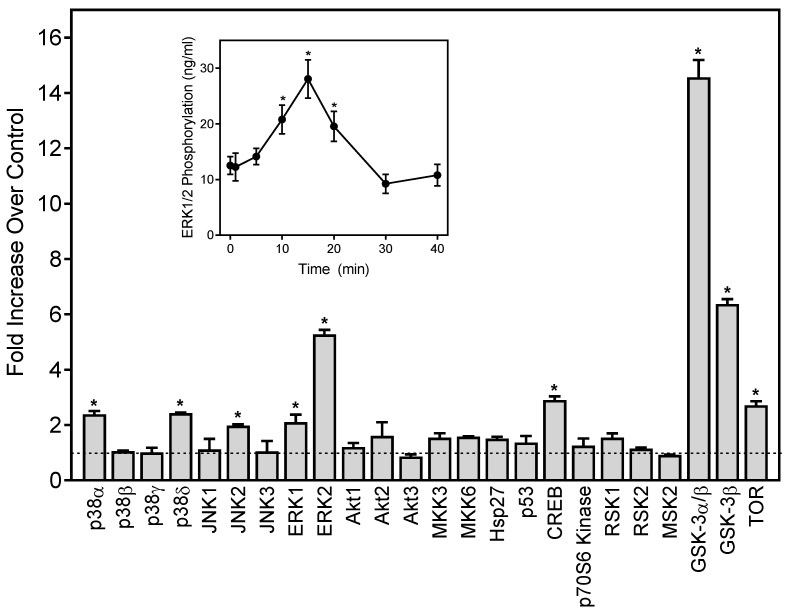
Effect of 1,3-dithiane on protein kinase phosphorylation in differentiated HL60 cells. The cells were treated for 15 min with 1,3-dithiane (500 μM), and the levels of protein phosphorylation in cell lysates were evaluated using a human phospho-kinase array. Statistically significant differences (* *p* < 0.05) between 1% DMSO (control, dash line) and 1,3-dithiane-treated cells are indicated. The data are presented as mean ± S.D. of duplicate samples from one experiment that is representative of two independent experiments. Inset: Effect of 1,3-dithiane on extracellular signal-regulated kinase 1/2 (ERK1/2) phosphorylation in differentiated HL60 cells. The cells were treated with 1,3-dithiane (500 µM) for the indicated times, and the levels of ERK phosphorylation were evaluated using an ELISA for human phospho-ERK1/2. The data are presented as mean ± S.D. of triplicate samples. Statistically significant differences between cells treated for 0 min (* *p* < 0.01) are indicated. The results shown are representative of three independent experiments.

Neutrophil functional response depends on multiple signaling pathways, including extracellular-signal regulated kinase (ERK), which is one of the major mitogen-activated protein kinases (MAPKs) [54,55]. To evaluate the effects of 1,3-dithiane on the activation of a number of signaling kinases, including the three major MAPKs, ERK1/ERK2, c-Jun N-terminal kinases (JNK 1–3), four p38 MAPK isoforms (α, β, δ, and γ), and other intracellular kinases such as mitogen- and stress-activated kinase 2 (MSK2), mammalian target of rapamycin (mTOR), cAMP response element-binding (CREB) protein, heat shock protein 27 (Hsp27), p53, Akt, glycogen synthase kinase (GSK-3), p90 ribosomal S6 kinase (RSK)1/2, MAP kinase kinases (MKK3 and MKK6), and p70 S6 kinase 1 (p70S6K1), we evaluated the global intracellular kinase signaling response to 1,3-dithiane in differentiated neutrophil-like HL60 cells with a human phospho-MAPK array. Differentiated HL60 cells were pretreated for 15 min with 1,3-dithiane (500 µM) or 1% DMSO (control), and the levels of protein phosphorylation in the cell lysates were evaluated. As shown in Figure 3, treatment with 1,3-dithiane significantly (≥two-fold) increased the phosphorylation of p38α (Thr180/Tyr182; fold increase (FI) = 2.3), p38δ (Thr180/Tyr182; FI = 2.3), JNK2 (Thr183/Tyr185, FI = 2.0), ERK1 (Thr202/Tyr204, FI = 2.1), ERK2 (Thr185/Tyr187; FI = 5.5), CREB (Ser133; FI = 2.9), GSK-3α/β (Ser21/Ser9; FI = 14.8), GSK-3β (Ser9; FI = 6.4), and mTOR (Ser2448, FI = 2.7).

Activation of ERK1/2 phosphorylation by 1,3-dithiane was one of the primary responses observed in our kinase array (Figure 3). Thus, we further characterized the time-dependent modulation of this response and found that activation of ERK1/2 phosphorylation was maximal after a 15-min treatment with 1,3-dithiane (Figure 3, Inset).

Although various EOs and their major compounds have been reported to modulate ROS production and Ca^2+^ mobilization in human neutrophils, including oils with a high content of organosulfur compounds [11,30,31], the current study is the first to report the biological effects of 1,3-dithiane, a small molecule organosulfur compound identified in *A. sativum* and boiled beef extracts [13,39,47]. Here, we found that 1,3-dithiane stimulates ROS production by human neutrophils, and this response was inhibited by specific inhibitors of PI3Kβ, γ, and δ. Activation of PI3K signaling usually occurs following the stimulation of receptor tyrosine kinases or G-protein-coupled receptors (GPCRs) [56]. We can exclude a stimulatory effect of 1,3-dithiane on several GPCRs, including *N*-formyl peptide receptors (FPRs) 1 and 2, C5a receptor, and chemokine receptors CXCR1/2, as this compound did not stimulate neutrophil Ca^2+^ mobilization (Table 2). PI3Ks phosphorylate phosphatidylinositol-(4,5) bisphosphate to form phosphatidylinositol (3,4,5)-trisphosphate (PIP3), and the accumulation of PIP3 facilitates the localization of Akt to the plasma membrane and its subsequent activation following phosphorylation. PI3K/Akt signaling regulates many of the molecular mechanisms contributing to increased ROS production through the phosphorylation and activation of NADPH oxidase subunits [56]. In this study, we also established that 1,3-dithiane activates ERK signaling, which in turn phosphorylates CREB, which has been reported for other bioactive molecules, such as lysophosphatidic acid [57]. Indeed, we found that 1,3-dithiane stimulated the phosphorylation of CREB and GSK-3α/β in differentiated HL60 cells. It should be noted that PI3K generation of PIP3 could directly or indirectly affect CREB phosphorylation [58].

Using the SwissADME online tool, we found that 1,3- and 1,4-dithianes have similar bioavailabilities (not shown) and physicochemical properties (Table 3). Numerous bioavailability parameters for these compounds are very close to each other, except lipophilicities (consensus Log P_o/w_ values of 1.86 and 1.70, respectively). 

To estimate the reactivity of 1,3- and 1,4-dithiane in biological environments, we performed a theoretical study of the molecules using the density functional theory (DFT) method. For geometry optimization and electronic structure calculations, the BP86 functional [59] and def2-TZVPP basis set [60] were used. At this level of theory, high-quality results can be obtained for conformations, energies, and electronic properties of organic compounds [61]. The values of Gibbs energies indicate that the two dithianes are thermodynamically most stable in the “chair” conformation, for which we have analyzed their calculated properties. We found that 1,3- and 1,4-dithiane differ markedly in their electronic structures. In contrast to the zero polarity of 1,4-dithiane, the calculated dipole moment equals 2.11 D for 1,3-dithiane, which is very close to the experimental values of 2.13 and 2.09 D measured in tetrachloromethane and benzene solutions, respectively [62].

**Figure 4:**
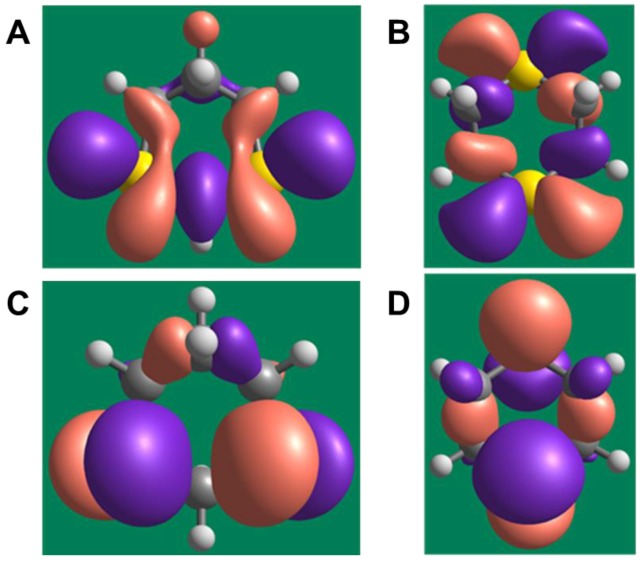
Isosurfaces of frontier orbitals in 1,3- and 1,4-dithiane. (**A**) and (**B**) represent lowest unoccupied molecular orbitals (LUMOs) of 1,3- and 1,4-dithiane, respectively. (**C**) and (**D**) represent highest occupied molecular orbitals (HOMOs) of 1,3- and 1,4-dithiane, respectively. A contour value of 0.03 for the isosurfaces was used.

The lowest unoccupied molecular orbital (LUMO) energies were equal to −0.702 eV (1,3-dithiane) and −0.280 eV (1,4-dithiane). The difference in LUMO energies of >0.4 eV is substantial and indicates that 1,3-dithiane is a better electron acceptor and can relatively easily form an anion-radical via one-electron reduction. The highest occupied molecular orbitals (HOMOs) have a smaller energy difference (−5.162 and −5.252 eV for 1,3- dithiane and 1,4-dithiane, respectively). Nevertheless, the HOMO of 1,3-dithiane is higher in energy (less negative value) than that of 1,4-dithiane. This reflects a higher tendency to oxidation, in accordance with previous results [48], and the greater ability of 1,3-dithiane to form complexes with transition metals. It should also be noted that HOMOs in both dithianes are localized mainly on sulfur atoms participating in complexation with metal ions (see the isosurfaces of frontier orbitals in Figure 4).

Chemical hardness, calculated as a HOMO–LUMO energy gap [63], is equal to 4.460 eV for 1,3-dithiane and 4.972 eV for 1,4-dithiane, also indicating the higher chemical reactivity of 1,3-dithiane, including reactivity to possible biotargets. Thus, the characteristics of the electronic structure obtained from the DFT calculations differ noticeably between the two dithianes, which may explain their differences in biological activity. Further studies are needed to examine the molecular targets of 1,3-dithiane or its biotransformation products that could be involved in phagocyte stimulatory effects. 

Various disulfides and trisulfides derived from garlic and onion have been shown to modulate immune functions and inflammation [32,64]. Previous studies demonstrated that some allyl-containing garlic-derived organosulfur compounds, including diallyl sulfide, diallyl disulfide, and diallyl trisulfide, can induce Ca^2+^ flux in several cell types, such as human glioblastoma cells, human colon cancer cells, and Madin–Darby canine kidney renal tubular cells [65,66,67]. Moreover, diallyl disulfide activated transient receptor potential (TRP) A1 channels [68]. However, there are no reports on the neutrophil modulatory effects of pure organosulfur compounds. Here, we show that several pure organosulfur compounds, including allyl propyl disulfide, dipropyl disulfide, diallyl disulfide, and AITC activate neutrophils, resulting in increased intracellular Ca^2+^, with the most potent being AITC. Previously, it was reported that some organosulfur compounds with an allyl group (diallyl sulfide, diallyl disulfide, diallyl trisulfide, and AITC) and ajoene were able to activate TRPA1 and/or TRPV1 channels [68,69,70,71,72,73]. TRPA1 is also activated by allicin from garlic and S-alkyl-S-alkenyldisulfides from asafoetida [68,69,71]. Various TRP channels are functionally expressed in neutrophils, and a transient elevation in intracellular [Ca^2+^]_i_ through activation of these channels can regulate various aspects of inflammatory and immune responses [74]. Human neutrophils only express members of the TRPC, TRPM, and TRPV channels [75]. However, SB 366791, a specific TRPV1 antagonist, did not inhibit Ca^2+^ flux in neutrophils induced by our active organosulfur compounds (diallyl sulfide, diallyl disulfide, diallyl trisulfide, and AITC), indicating that TRPV1 is likely not involved in this response (data not shown). Thus, further studies are needed to examine the specific mechanisms and targets involved. 

In conclusion, our data provide a molecular basis to explain at least part of the beneficial therapeutic effects of mustard oil, garlic EO, and garlic extracts and suggest that neutrophil stimulation by organosulfur components from *Allium* spp. might enhance resistance to infection. Future studies are planned to investigate the therapeutic potential of organosulfur compounds for various disorders with immune and/or inflammatory mechanisms.

## 3. Materials and Methods

### 3.1. Screening Compounds and Garlic EO

Allyl methyl sulfide was purchased from Toronto Research Company (Toronto, ON, Canada). Dipropyl trisulfide was from Combi-Blocks (San Diego, CA, USA), and (+)-l-alliin was from MP Biomedicals (Solon, OH, USA). Ajoene was from Santa Cruz Biotechnology (Santa Cruz, CA, USA). Allyl propyl sulfide, allyl methyl disulfide, and allyl propyl disulfide were purchased from TCI America (Portland, OR, USA). AITC, cyclopentanethiol, diallyl sulfide, diallyl disulfide, dimethyl disulfide, dimethyl trisulfide, dipropyl sulfide, 2,5-dimethylthiophene, 1,3-dithiane, and dipropyl disulfide were from Alfa Aesar (Haverhill, MA, USA). 1,4-Dithiane, 1,3-dithiane-1-oxide, and methyl propyl disulfide were from Sigma-Aldrich Chemical Co. (St. Louis, MO, USA). Diallyl trisulfide and *N*-acetyl-*S*-allyl-L-cysteine were from Cayman Europe (Tallinn, Estonia). Allicin and *S*-allyl-L-cysteine were from AK Scientific Inc. (Union City, CA, USA). Garlic EO was obtained from Silky Scents, LLC (Corona, CA, USA). 

### 3.2. Materials for Biological Assays

Dimethyl sulfoxide (DMSO), N-formyl-methionine-leucine-phenylalanine (*f*MLF), PMA, SOD from bovine erythrocytes, and Histopaque 1077 were purchased from Sigma-Aldrich Chemical Co. (St. Louis, MO, USA). Fluo-4AM was from Invitrogen (Carlsbad, CA, USA). 8-Amino-5-chloro-7-phenylpyridol[3,4-d]pyridazine-1,4(2*H*,3*H*)-dione (L-012) was from Wako Chemicals (Richmond, VA, USA). PI3K inhibitors (PI 3065, TGX 221, AS 605240, and A66) and the specific TRPV1 antagonist SB 366791 were from Tocris Bioscience (Minneapolis, MN, USA). Fluo-4AM was from Invitrogen (Carlsbad, CA, USA). The penicillin–streptomycin solution was from Mediatech (Herndon, VA, USA). Fetal bovine serum was from Atlas Biologicals (Fort Collins, CO, USA). Hanks’ balanced salt solution (HBSS; 0.137 M NaCl, 5.4 mM KCl, 0.25 mM Na_2_HPO_4_, 0.44 mM KH_2_PO_4_, 4.2 mM NaHCO_3_, 5.56 mM glucose, and 10 mM HEPES, pH 7.4) was from Life Technologies (Grand Island, NY, USA). HBSS without Ca^2+^ and Mg^2+^ is designated as HBSS^−^; HBSS containing 1.3 mM CaCl_2_ and 1.0 mM MgSO_4_ is designated as HBSS^+^.

### 3.3. Isolation of Human Neutrophils

For the isolation of human neutrophils, blood was collected from healthy donors in accordance with a protocol approved by the Institutional Review Board at Montana State University (Protocol #MQ041017). Neutrophils were purified from the blood using dextran sedimentation, followed by Ficoll-Paque 1077 gradient separation and hypotonic lysis of red blood cells, as described previously [31]. Isolated neutrophils were washed twice and resuspended in HBSS^-^. Neutrophil preparations were routinely >95% pure, as determined by light microscopy, and >98% viable, as determined by trypan blue exclusion. Neutrophils were obtained from multiple donors (*n* = 8); however, the cells from different donors were never pooled together during experiments.

### 3.4. Cell Culture

Human promyelocytic leukemia HL60 cells were cultured in RPMI 1640 medium supplemented with 10% heat-inactivated fetal calf serum, 10 mM HEPES, 100 μg/mL streptomycin, and 100 U/mL penicillin. For the differentiation of HL60 into neutrophil-like cells, DMSO was added to a final concentration of 1.2%, and the cells were cultured for 6 days. Differentiation was monitored by a gain in responsiveness of the cells to *f*MLF by measuring *f*MLF-induced Ca^2+^ mobilization (data not shown).

### 3.5. Ca^2+^ Mobilization Assay

Changes in neutrophil intracellular Ca^2+^ concentrations ([Ca^2+^]_i_) were measured with a FlexStation 3 scanning fluorometer (Molecular Devices, Sunnyvale, CA, USA). Briefly, human neutrophils were suspended in HBSS^-^, loaded with Fluo-4AM at a final concentration of 1.25 μg/mL, and incubated for 30 min in the dark at 37 °C. After dye loading, the cells were washed with HBSS^–^, resuspended in HBSS^+^, separated into aliquots, and aliquoted into the wells of flat-bottom, half-area well black microtiter plates (2 × 10^5^ cells/well). Test compounds diluted in DMSO were added to the wells (final concentration of DMSO was 1%), and the changes in fluorescence were monitored (λ_ex_ = 485 nm, λ_em_ = 538 nm) every 5 s for 240 s at room temperature. The maximum change in fluorescence observed after subtracting the background signal from DMSO-treated cells was used to determine the agonist response. Antagonist activity was evaluated after a 30 min pretreatment with test compounds at room temperature, followed by the addition of the peptide agonist (5 nM *f*MLF). Maximum change in fluorescence during the first 3 min, expressed in arbitrary units over baseline, was used to determine a response. Responses for test compounds were normalized to the response induced by 5 nM *f*MLF, which was assigned a value of 100%. Curve fitting (5–6 points) and calculation of median effective inhibitory concentrations (EC_50_ or IC_50_) were performed by nonlinear regression analysis of the dose–response curves generated using Prism 8 (GraphPad Software, Inc., San Diego, CA, USA), as described previously [30].

### 3.6. ROS Production Assay

ROS production was determined by monitoring L-012-enhanced CL, which represents a sensitive and reliable method for detecting ROS production [76]. Human neutrophils were resuspended at 10^6^ cells/mL in HBSS^+^ supplemented with 40 µM L-012. Cells (100 µL) were aliquoted into wells of 96-well flat-bottomed white microtiter plates containing test compounds (final DMSO concentration of 1%). Neutrophils were stimulated by the application of 100 nM PMA. Luminescence was monitored for 60 min (2-min intervals) at 37 °C using a Fluroscan Ascent FL microtiter plate reader (Thermo Electron, Waltham, MA, USA). To calculate IC_50_ values, individual ROS responses were normalized to the maximal response in a given experiment, which was assigned a value of 100%. Curve fitting (at least five or six points) and calculation of IC_50_ values were performed by nonlinear regression analysis of the dose–response curves generated using Prism 8 (GraphPad Software, Inc., San Diego, CA, USA).

### 3.7. Protein Kinase Array

Analysis of the phosphorylation profiles of MAPKs and related kinases and their substrates was performed using a human phospho-kinase array kit, Proteome Profiler (R&D Systems, Minneapolis, MN, USA). The array simultaneously detects 24 kinase phosphorylation sites, including MAPKs (ERK1/2, c-Jun N-terminal kinases (JNK 1–3), p38 isoforms α, β, γ, σ), MSK2, MKK3, MKK6, hsp27, p53, mTOR, Akt 1–3, GSK-3α/β, RSK 1/2, p70S6K, and CREB. For the analysis, differentiated neutrophil-like HL60 cells were incubated for 15 min with 500 µM of 1,3-dithiane or negative control (1% DMSO) at 37 °C. The cells were then lysed, and the arrays were incubated overnight at 4 °C with lysates obtained from 10^7^ cells for each sample. The arrays were washed three times with 20 mL of the wash buffer, followed by incubation for 2 h with the detection antibody cocktail containing phospho-site-specific biotinylated antibodies. The wash steps were repeated, the arrays were exposed to chemiluminescent reagents, and the signal was captured with an Alpha Innotech FluorChem FC2 imaging system.

### 3.8. ERK1/2 Enzyme-Linked Immunosorbent Assay (ELISA)

Differentiated HL60 cells were incubated for various times (0, 1, 5, 10, 15, 20, 30, and 40 min) with 500 µM of 1,3-dithiane or negative control (1% DMSO) at 37 °C. The cells were lysed with lysis buffer (R&D Systems), and the levels of phosphorylated ERK1/2 were measured in the cell lysates using an ELISA kit for human phospho-ERK1 (Thr202/Tyr204)/ERK2 (Thr185/Tyr187) (R&D Systems). The concentrations of phospho-ERK1/2 in the cell lysates were determined using a calibration curve with recombinant human phospho-ERK2 (Thr85/Tyr187).

### 3.9. Analysis of 1,3-Dithiane Biotransformation

Biotransformation of 1,3-dithiane in culture medium from HL60 cells was evaluated by GC-MS analysis. HL60 cells were cultured in RPMI 1640 medium supplemented with 10 mM HEPES and 4 mM 1,3-dithiane for 0, 1, and 5 h at 37 °C. The samples were extracted with methanol for 4 h and then centrifugated. GC-MS analysis was performed on an Agilent 7890A GC with a 5890A mass-selective detector (MSD) system. A Restek Rtx-VMS column (30 m × 0.25 mm, 1.40 μm film thickness) was used with He as the carrier gas (2.0 mL/min). GC oven temperature was kept at 30 °C for 1 min, increased to 250 °C at a rate of 11 °C/min, and kept constant at 250 °C for 4 min. The split ratio was adjusted to 10:1, and the injector temperature was 250 °C. Commercial 1,3-dithiane-1-oxide was used as a reference. The MS parameters were as follows: transfer line temperature was set at 250 °C, source temperature at 230 °C and quad temperature at 150 °C. Acquisition range was set between 30 and 550 amu at a scan rate of 5.2 scans/sec. Standard 1,3-dithiane and 1,3-dithiane-1-oxide were injected to identify the retention time. Under these conditions, 1,3-dithane eluted at 15.1 min and 1,3-dithiane-1-oxide eluted at 21.6 min. The corresponding M^+^ ions of 1,3-dithiane and 1,3-dithiane-1-oxide were seen at 120.00 and 136.00 *m*/*z*, respectively.

### 3.10. Molecular Modeling

The physicochemical properties and absorption, distribution, metabolism, and excretion (ADME) parameters were computed using SwissADME (http://www.swissadme.ch).

DFT calculations were performed with the ORCA 4.1.1 program (Max Planck Institute fuer Kohlenforschung, Muelheim/Ruhr, Germany, December 2018) on a computer operating under Windows Server 2016 (16 × 2.4 GHz CPU, 16 Gb RAM). The BP86 functional [59], def2-TZVPP orbital basis set [60], resolution of identity (RI) approximation with def2/J auxiliary basis set [61], and D3BJ dispersion correction [77] were applied. The attainment of real energy minima on geometry optimizations in vacuo was confirmed by frequency calculations. The obtained results were visualized and analyzed with the use of Chemcraft graphical software for the visualization of quantum chemistry computations (https://www.chemcraftprog.com).

## Figures and Tables

**Table 1:** Selected organosulfur compounds identified in essential oils (EOs) and extracts from *Allium* spp. and mustard.

Compound Name	Chemical Structure	Plant Species	% ^a^	References
Allyl methyl sulfide	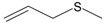	*Allium ursinum*	0.0–0.1	[20]
		*Allium sativum*	tr	[13]
Dipropyl sulfide	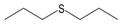	*Allium sativum*	n.d.	[37]
Allyl propyl sulfide	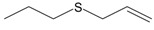	*Allium ursinum*	tr	[20]
		*Allium sativum*	0.0–0.1	[38]
Diallyl sulfide	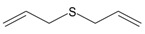	*Allium sativum*	1.6–9.5	[38,39,40]
		*Allium ursinum*	0.1–0.3	[20]
Dimethyl disulfide	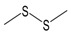	*Allium sativum*	0.4–1.4	[38]
		*Allium ursinum*	0.7–2.3	[20]
Methyl propyl disulfide	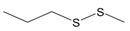	*Allium sativum*	tr	[13]
Dipropyl disulfide	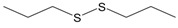	*Allium porrum*	29.8	[40]
		*Allium ursinum*	0.0–0.3	[20]
Allyl methyl disulfide	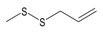	*Allium sativum*	4.4–8.3	[38]
		*Allium ursinum*	1.1–18.9	[20]
Allyl propyl disulfide	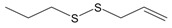	*Allium sativum*	3.1	[39]
Diallyl disulfide	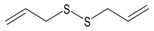	*Allium ursinum*	9.9–20.7	[20]
		*Allium sativum*	20.8–29.1	[38,39,40]
Dimethyl trisulfide	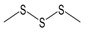	*Allium sativum*	1.3–2.9	[38]
		*Allium ursinum*	1.1–7.5	[20]
Dipropyl trisulfide	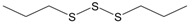	*Allium ursinum*	tr	[20]
Diallyl trisulfide	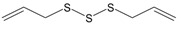	*Allium sativum*	16.8–50.4	[38,39,40]
		*Allium ursinum*	5.2–19.6	[20]
Allyl isothiocyanate (AITC)	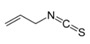	*Sinapis alba* (mustard seed)	71.1	[41]
Allicin	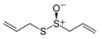	*Allium sativum*	(3 mg/g) ^b^	[42]
2,5-Dimethylthiophene	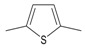	*Allium fistulosum*	tr	[43]
1,3-Dithiane	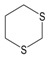	*Allium sativum*	2.1	[13,39]

^a^ Percentage composition is indicated for EOs. ^b^ Based on the weight of dry plant material.

**Table 2:** Effect of the selected organosulfur compounds and garlic EO on human neutrophil functional responses.

Compound Common Name	Ca^2+^ Flux		Spontaneous CL	Stimulated CL
	Activation EC_50_ (µM)	Inhibition IC_50_ (µM)	Inhibition IC_50_ (µM)	
Allyl methyl sulfide	N.A.	N.A.	N.A.	N.A.
Dipropyl sulfide	N.A.	N.A.	N.A.	N.A.
Allyl propyl sulfide	N.A.	N.A.	N.A.	N.A.
Diallyl sulfide	N.A.	N.A.	N.A.	N.A.
Dimethyl disulfide	N.A.	N.A.	N.A.	N.A.
Methyl propyl disulfide	N.A.	30.2 ± 4.6	N.A.	N.A.
Dipropyl disulfide	13.1 ± 3.4	29.1 ± 4.8	N.A.	N.A.
Allyl methyl disulfide	N.A.	N.A.	N.A.	N.A.
Allyl propyl disulfide	22.5 ± 6.2	18.6 ± 4.1	N.A.	N.A.
Diallyl disulfide	9.8 ± 2.1	22.1 ± 3.7	N.A.	N.A.
Dimethyl trisulfide	N.A.	N.A.	N.A.	N.A.
Dipropyl trisulfide	N.A.	N.A.	N.A.	N.A.
Diallyl trisulfide	N.A.	N.A.	17.7 ± 3.3	39.0 ± 5.2
Allyl isothiocyanate (AITC)	7.9 ± 1.8	20.8 ± 4.3	N.A.	N.A.
Allicin	N.A.	30.7 ± 5.1	1.5 ± 0.3	4.4 ± 0.6
Ajoene	N.A.	N.A.	5.5 ± 1.4	22.1 ± 4.1
Alliin	N.A.	N.A.	N.A.	17.5 ± 2.8
*N*-acetyl-*S*-allyl-L-cysteine	N.A.	N.A.	N.A.	9.5 ± 2.1
*S*-allyl-L-cysteine	N.A.	N.A.	N.A.	10.9 ± 0.9
2,5-Dimethylthiophene	N.A.	N.A.	N.A.	N.A.
Cyclopentanethiol	N.A.	N.A.	N.A.	N.A.
1,3-Dithiane	N.A.	N.A.	*	
	**(µg/mL)**			
Garlic EO	34.9 ± 2.8	14.7 ± 2.4	5.5 ± 1.2	3.7 ± 0.8

**Table 3:** Physicochemical properties of 1,3- and 1,4-dithianes.

Properties	1,3-Dithiane	1,4-Dithiane
2D Structure	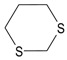	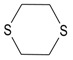
Formula	C_4_H_8_S_2_	
Molecular weight	120.24 g/mol	
Num. heavy atoms	6	
Fraction Csp3	1.00	
Num. rotatable bonds	0	
Num. H-bond acceptors	0	
Num. H-bond donors	0	
Molar refractivity	34.41	
Topological polar surface area	50.60 Å²	
Lipophilicity (consensus Log P_o/w_)	1.86	1.70

## Abbreviations

ADME	absorption, distribution, metabolism, and excretion
AITC	allyl isothiocyanate
CL	chemiluminescent
CREB	cAMP response element binding
DFT	density functional theory
DMSO	dimethyl sulfoxide
EI	electron impact
ELISA	enzyme-linked immunosorbent assay
EO	essential oil
ERK1/2	extracellular signal–regulated kinase 1/2
GC-MS	gas chromatography–mass spectrometry
GPCR	G-protein-coupled receptor
GSK-3α/β	glycogen synthase kinase 3 α/β
HOMO	highest occupied molecular orbital
hsp27	heat shock protein 27
JNK	c-Jun N-terminal kinases
LUMO	lowest unoccupied molecular orbital
MAPK	mitogen activated protein kinase
MKK	MAP kinase kinase
MSK	mitogen- and stress-activated kinase
mTOR	mammalian target of rapamycin
NADPH	reduced nicotinamide adenine dinucleotide phosphate
NO	nitric oxide
p70S6K1	p70 S6 kinase 1
PIP3	phosphatidylinositol (3,4,5)-trisphosphate
PMA	phorbol-12-myristate-13-acetate
PI3K	phosphatidylinositol-3 kinase
PIP3	phosphatidylinositol (3,4,5)-trisphosphate
ROS	reactive oxygen species
RSK	p90 ribosomal S6 kinase
SOD	superoxide dismutase
TRP	transient receptor potential
